# Growing Global Research Interest in Antimicrobial Peptides for Caries Management: A Bibliometric Analysis

**DOI:** 10.3390/jfb13040210

**Published:** 2022-10-29

**Authors:** Olivia Lili Zhang, John Yun Niu, Iris Xiaoxue Yin, Ollie Yiru Yu, May Lei Mei, Chun Hung Chu

**Affiliations:** 1 Faculty of Dentistry, The University of Hong Kong, Hong Kong, China; 2 Faculty of Dentistry, The University of Otago, Dunedin 9054, New Zealand

**Keywords:** antimicrobial, caries, peptides, prevention, remineralisation

## Abstract

**Objective:** Researchers are studying the use of antimicrobial peptides as functional biomaterials to prevent and treat dental caries. This study aims to investigate the global research interest in antimicrobial peptides for caries management. **Methods:** Two independent investigators systematically searched with keywords (‘Caries’ OR ‘Dental caries’) AND (‘Antimicrobial peptide’ OR ‘AMP’ OR ‘Statherin’ OR ‘Histatin’ OR ‘Defensin’ OR ‘Cathelicidin’) on Web of Science, PubMed and Scopus. They removed duplicate publications and screened the titles and abstracts to identify relevant publications. The included publications were summarized and classified as laboratory studies, clinical trials or reviews. The citation count and citation density of the three publication types were compared using a one-way analysis of variance. The publications’ bibliometric data were analyzed using the Bibliometrix program. **Results:** This study included 163 publications with 115 laboratory studies (71%), 29 clinical trials (18%) and 19 reviews (11%). The number of publications per year have increased steadily since 2002. The citation densities (mean ± SD) of laboratory study publications (3.67 ± 2.73) and clinical trial publications (2.63 ± 1.85) were less than that of review articles (5.79 ± 1.27) (*p* = 0.002). The three publication types had no significant difference in citation count (*p* = 0.54). Most publications (79%, 129/163) reported the development of a novel antimicrobial peptide. China (52/163, 32%) and the US (29/163, 18%) contributed to 50% (81/163) of the publications. Conclusion: This bibliometric analysis identified an increasing trend in global interest in antimicrobial peptides for caries management since 2002. The main research topic was the development of novel antimicrobial peptides. Most publications were laboratory studies, as were the three publications with the highest citation counts. Laboratory studies had high citation counts, whereas reviews had high citation density.

## 1. Introduction

The application of antimicrobial peptides in managing various diseases has attracted significant interest from researchers over the past two decades [1]. In general, multicellular organisms produce antimicrobial peptides as an immune response. Antimicrobial peptides have a small molecular mass, between 1 and 5 kDa, and consist of 12–50 amino acid residues. They can form amphipathic structures in nonpolar solvents, which are essential for their antimicrobial properties; these structures are the typical secondary structures of antimicrobial peptides, including α-helical and β-sheet. In addition, antimicrobial peptides play an essential role as effectors or regulators of the innate immune system [2].

The mechanisms of antimicrobial peptides against microbes could be described as directly bactericidal, functioning by inducing pore formation on the cytoplasmic membrane or by splitting the microbes’ cytoplasmic membrane [3]. The antimicrobial peptide models attacking cytoplasmic membrane bilayers include ‘toroidal-pore’, ‘carpet’, ‘detergent-like’ or ‘barrel-stave’ mechanisms [4]. Because the positive peptides only attach to negatively charged bacteria, antimicrobial peptides have selective toxicity without damaging the host’s tissues. In addition, resistance mutations are not easily induced because antimicrobial peptides usually attack multiple hydrophobic and polyanionic bacterial targets [5]. Thus, antimicrobial peptides are a potential alternative to traditional antimicrobial agents.

In dentistry, antimicrobial peptides play multifaceted roles in various oral diseases [1]. Natural antimicrobial peptides can kill oral pathogens and modulate innate and acquired immunity [6]. The level of natural antimicrobial peptides in saliva and gingival crevicular fluid can indicate caries risk [7]. Antimicrobial peptides’ flexible and various structures make possible functional modifications of antimicrobial peptides to extend their potential applications. In addition, researchers believe that novel synthetic antimicrobial peptides can be safely used and promote oral health and overall health [8]. Moreover, dental caries develops due to bacteria metabolising sucrose to produce acid that demineralises the hard tissues of the teeth [9]. Thus, researchers are developing novel antimicrobial peptides with ideal antimicrobial properties as anti-caries agents. For example, synthetic antimicrobial peptides Amyl-1–18 from rice [10] and HBD3-C15 [11] from human beta-defensin (hBD)-3 showed bactericidal activity against *Streptococcus mutans*. C16G2 [12] is a specifically targeted antimicrobial peptide which can selectively and quickly kill *S. mutans.* SspB (390–T400K–402) [13] is a competitive peptide that prevents the adhesion of *S. mutans* to the salivary pellicle on hydroxyapatite. in addition, GA-KR12, with antimicrobial and mineralising properties, is a novel antimicrobial peptide made by fusing the broad-spectrum antimicrobial peptide KR12 and the mineralising molecule gallic acid [14]. 

Although different kinds of bioactive materials could be used in caries management [15], not many have been developed into clinical applications, due to different problems. For example, using nanoparticles based on metal or metallic oxide for caries management is being extensively researched [16]. However, the toxicity of nanoparticles to mammalian cells concerns researchers [17]. Some other research focuses on the use of natural phytochemicals for caries management. However, the antibacterial potential of phytochemicals is limited [18]. An ideal anti-caries agent, silver diamine fluoride, has been demonstrated to effectively prevent and arrest dental caries [19]. The major disadvantage of silver diamine fluoride is dental staining. In comparison, antimicrobial peptides have selective toxicities, a low chance of inducing resistance [20] and ideal antimicrobial properties [21,22]. In sum, antimicrobial peptides are potential anti-caries agents and research hotspots [8].

Bibliometric data is quantitative data from published scientific works. The quantitative indices of bibliometric analyses include citation counts; citation density by year; and lists of works by author, country and paper types [23]. A computer program can be used to extract these data from electronic databases [23]. These data, among other purposes, can be used for obtaining an overview of current research to assess the scientific impact, detect the top area of a research field, explore the research trends and discover the leading researchers and institutions [23]. To date, bibliometric analyses have been widely used in various disciplines, including dentistry, to analyse research trends [24,25]. Although attention to antimicrobial peptides for caries management is increasing [8], there is no bibliometric study on this topic. The objective of this bibliometric study is to explore and quantify the global research interest in antimicrobial peptides for caries management.

## 2. Materials and Methods

### 2.1. Search Strategy

**Figure 1:**
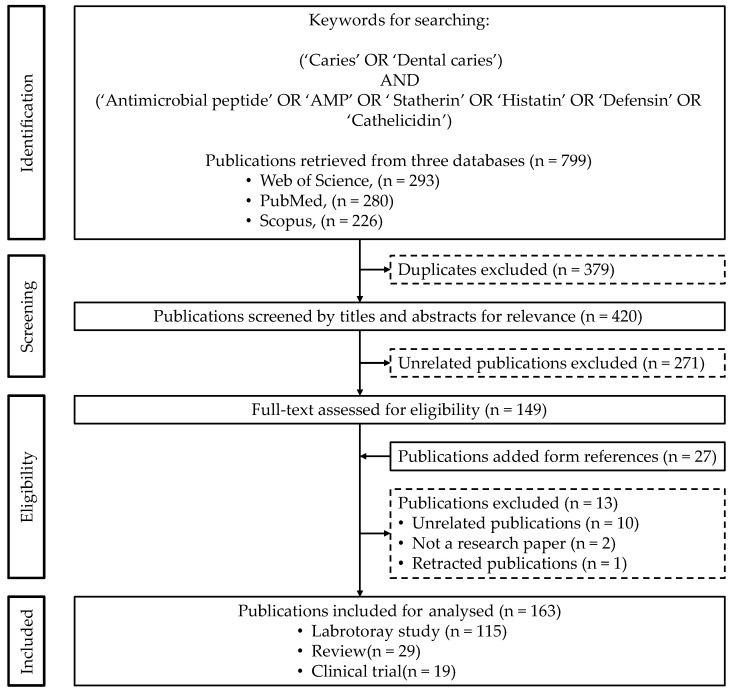
Flowchart of the study.

In August 2022, a literature search was conducted on the Web of Science (https://www.webofscience.com/, accessed on 1 August 2022), PubMed (https://pubmed.ncbi.nlm.nih.gov/, accessed on 1 August 2022) and Scopus (https://www.scopus.com/, accessed on 1 August 2022) to identify publications on antimicrobial peptides for caries management. The search keywords were (Caries OR Dental caries) AND (Antimicrobial peptide OR AMP OR Statherin OR Histatin OR Defensin OR Cathelicidin). The full search strategy on Web of Science was “TS = ((‘Antimicrobial peptide‘ OR ‘AMP’ OR ‘Cathelicidin’ OR ‘Defensin’ OR ‘Histatin’ OR ‘Statherin’) AND (‘Caries’ OR ‘Dental caries’))”. The full search strategy on PubMed was “(‘Antimicrobial peptide‘ OR ‘AMP’ OR ‘Cathelicidin’ OR ‘Defensin’ OR ‘Histatin’ OR ‘Statherin’) AND (‘Caries’ OR ‘Dental caries’)”. In addition, the full search strategy on Scopus was “TITLE-ABS-KEY ((“antimicrobial” AND “peptide” OR “amp” OR “cathelicidin” OR “defensin” OR “histatin” OR “statherin”) AND (“caries” OR “dental caries”))”. Figure 1 shows the flowchart of this study.

### 2.2. Study Selection

Two independent investigators (I.X.Y. and O.L.Z.) found and removed duplicate publications. Then, they screened titles and abstracts to exclude unrelated publications. After the first exclusion round, they retrieved the full texts and selected publications on antimicrobial peptides for caries management. In addition, the reference lists were manually screened to identify potentially missed publications. The inclusion criteria were the journal papers on the use of antimicrobial peptides for caries management. The exclusion criteria included: (1) a publication did not relate to dental caries and/or antimicrobial peptides, (2) a publication was not a research paper (e.g., book chapters, editorials, letters, communication and news) or (3) a publication had been retracted. Finally, the two investigators had an in-depth discussion to achieve agreement on the list of included publications. 

### 2.3. Data Management and Extraction

The full record of included publications was obtained from Web of Science, PubMed and Scopus. All the data were locally preserved. The R package program Biblioshiny (https://www.bibliometrix.org, accessed on 1 August 2022) was used to extract and analyse the bibliometric data [11]. Keywords, publication type, citation count, citation density (the average number of citations per year: citation count/(2022-published year)), publication number by year, journal of publication (type of publication, research theme and research focus) and authorship (authors’ countries and institutions) were extracted, analysed and visualised.

Bradford’s law and the Hirsch index (h-index) were used to assess the ‘core’ journals and their impact on the field of antimicrobial peptides for caries management. Bradford’s law describes the scatter of the number of papers published in a given field [26]. The ‘first zone’ Bradford defined included the ‘core’ journals of a particular field. The h-index was used to detect the impact of journals in this study [27]. In addition, international collaborations on antimicrobial peptides for caries management were quantified using the multiple-country publication (MCP) and single-country publication (SCP) scale. After being extracted, analysed and visualised, the secondary data were also locally preserved. 

### 2.4. Statistical Analysis

Differences between the citation data of the different publication types were assessed using one-way analysis of variance (ANOVA) using SPSS Statistics 20 (IBM Corporation, Somers, NY, USA). Multiple comparisons were also conducted using Bonferroni method. The cut-off level was set at 5% significance.

## 3. Results

The initial literature search revealed 799 publications (Figure 1) from Web of Science (*n* = 293), PubMed (*n* = 280) and Scopus (*n* = 226). Duplicate publications (*n* = 379) were removed, so that the remaining number was *n* = 420. After screening the title and abstracts, 271 publications unrelated to caries and/or antimicrobial peptides were excluded.

The reference lists of the remaining 149 publications were checked to find another 27 publications that might have met the inclusion criteria. Thus, the final number of texts subjected to screening for assessment reached 176 publications. Thirteen publications were excluded for the following reasons: unrelated to caries (*n* = 9), unrelated to antimicrobial peptides (*n* = 1), not a research paper (*n* = 2) and retracted paper (*n* = 1). Finally, the remaining 163 publications on antimicrobial peptides for caries management were included in this study (Supplementary Table S1).

**Figure 2:**
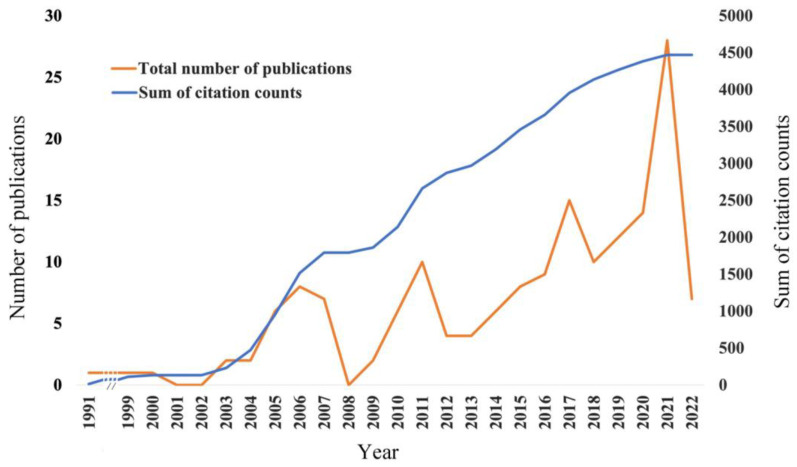
The number of publications per year and citation count per year on antimicrobial peptides for caries management from 1991 to 2022.

The number of publications on antimicrobial peptides for caries management per year has steadily increased since 2002 (Figure 2). Three peaks can be seen in Figure 2, 8 publications were published in 2006, 10 in 2010 and 15 in 2017. After 2018, the number of publications markedly increased from 10 in 2018 to 28 in 2021. In the first 6 months of 2022, 7 publications were published. The sum of citation counts also showed a steadily increasing trend since 2002 (Figure 2).

**Figure 3:**
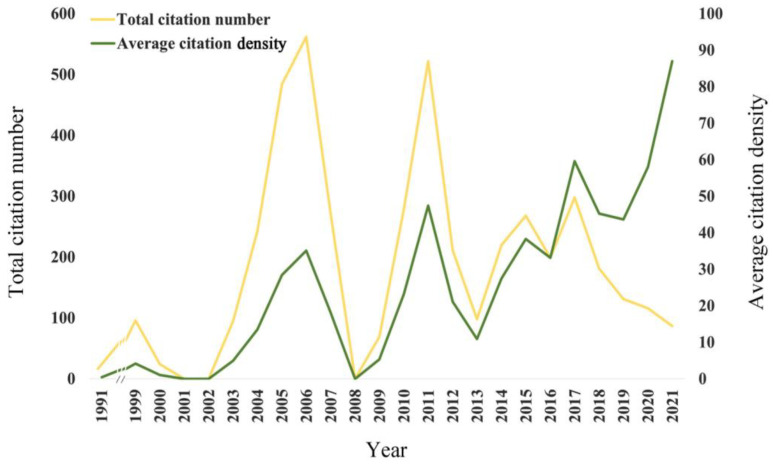
The citation count per year and citation density per year on antimicrobial peptides for caries management from 1991 to 2022.

Figure 3 shows the citation data of the included publications. The highest total citation count of publications (562) was in 2006.

*Citation density* = *Total citation number*/(2022 − *published year*)

The publication that obtained the highest citation count was a laboratory study on the antimicrobial properties of natural antimicrobial peptides HBD-2 and HBD-3 against *S. mutans* and other oral pathogens [28]. This was followed by Ouhara et al., 2005 [29], Wei et al., 2006 [30], Eckert et al., 2006 [31], and Batoni et al., 2011 [32]. Among the top five total citation counts publications, four were laboratory studies and one was a review (Table 1). The highest average citation density was in 2021 (87). The publication that obtained the highest citation density was a systematic review of the application of antimicrobial peptides for caries prevention and treatment [8]. This was followed by Qiu et al., 2020 [33], Mai et al., 2017 [3], Batoni et al., 2011 [32], and Xie et al., 2020 [34]. Among the top five citation density publications, four are reviews and one is a laboratory study (Table 1).

Table 2 shows that the main types of included publications were laboratory studies (*n* = 115), clinical trials (*n* = 19) and reviews (*n* = 29). The mean citation counts for laboratory studies, clinical trials and reviews were 29 ± 36, 22 ± 27 and 31 ± 34, respectively. There was no significant difference in the mean citation counts. The mean citation densities for laboratory studies, clinical trials and reviews were 3.67 ± 2.73, 2.63 ± 1.85 and 5.79 ± 1.27, respectively. Multiple comparisons using Bonferroni methods showed that the citation density of the reviews was significantly higher than that of laboratory studies and clinical trials (*p* = 0.002).

**Figure 4:**
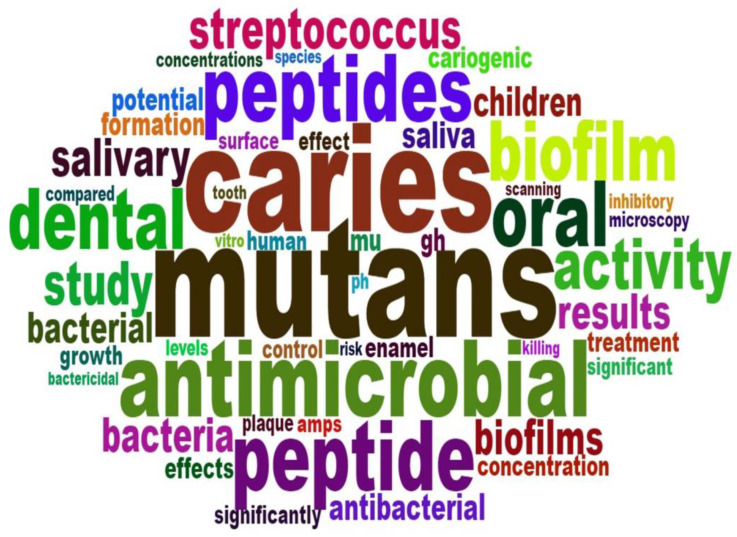
Word cloud of keywords of the publications on antimicrobial peptides for caries management in 2022.

For all the publications, the top keywords included ‘*Streptococcus mutans*’, ‘dental caries’, ‘antimicrobial peptides’, ‘biofilm’, ‘saliva’ and ‘mineralisation’. Figure 4 shows the word cloud of the included publications’ keywords.

In summary, the two main topics of these publications were novel antimicrobial peptides as anti-caries agents for caries management (*n* = 129) and natural antimicrobial peptides as biomarkers of caries risk (*n* = 34). Regarding the publications about novel antimicrobial peptides as anti-caries agents for caries management, the primary study objects were *S. mutans* in planktic or biofilm conditions.

Several publications (*n* = 33) also investigated the novel antimicrobial peptides’ mineralisation effect. For the publications about natural antimicrobial peptides as biomarkers of caries risk, the focus was the relationship between genetic polymorphisms of saliva antimicrobial peptides and the risk of caries.

Among the studies on novel antimicrobial peptides as anti-caries agents for caries management, most (92%) of the studies focused on the design, selection and application of antimicrobial peptides. Only ten of them (8%) tried to explain the mechanism. For example, Niu et al. used a transmission electron microscope to investigate the target for antimicrobial peptides attacking the bacterial membrane [14]. Wang et al. conducted quantitative real-time PCR to investigate the mechanism of antimicrobial peptides affecting bacterial metabolism [35]. Zhou et al. used molecular dynamic simulation analysis to investigate the interaction mechanisms between the peptide and tooth surface [36].

**Figure 5:**
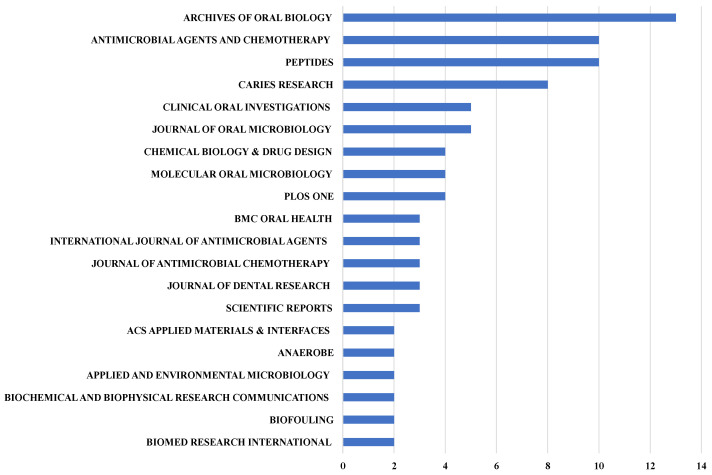
Twenty journals reporting antimicrobial peptides for caries management according to the number of publications.

The ‘core’ journals of the publications about antimicrobial peptides for caries management were *Archives of Oral Biology* (*n* = 12), *Antimicrobial Agents and Chemotherapy* (*n* = 10), *Peptides* (*n* = 10), *Caries Research* (*n* = 8), *Clinical Oral Investigations* (*n* = 5), *Journal of Oral Microbiology* (*n* = 5) and *Chemical Biology & Drug Design* (*n* = 4) (Figure 5).

**Figure 6:**
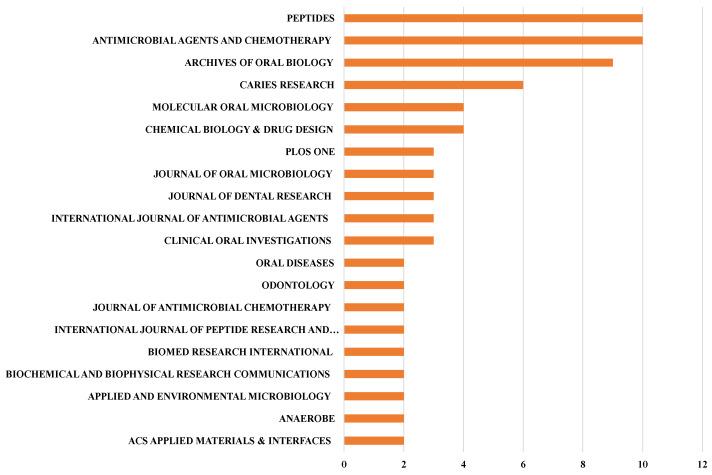
Twenty journals reporting antimicrobial peptides for caries management according to h-index of publications.

The ranking of the h-index of journals differed from the ranking of ‘core’ journals. The journals *Peptides* (h-index = 10) and *Antimicrobial Agents and Chemotherapy* (h-index = 10) had the highest scientific impact. They were followed by *Archives of Oral Biology* (h-index = 9), *Caries Research* (h-index = 6), *Molecular Oral Microbiology* (h-index = 4) and *Chemical Biology & Drug Design* (h-index = 4) (Figure 6). The h-index of *Clinical Oral Investigations* was only 3.

**Figure 7:**
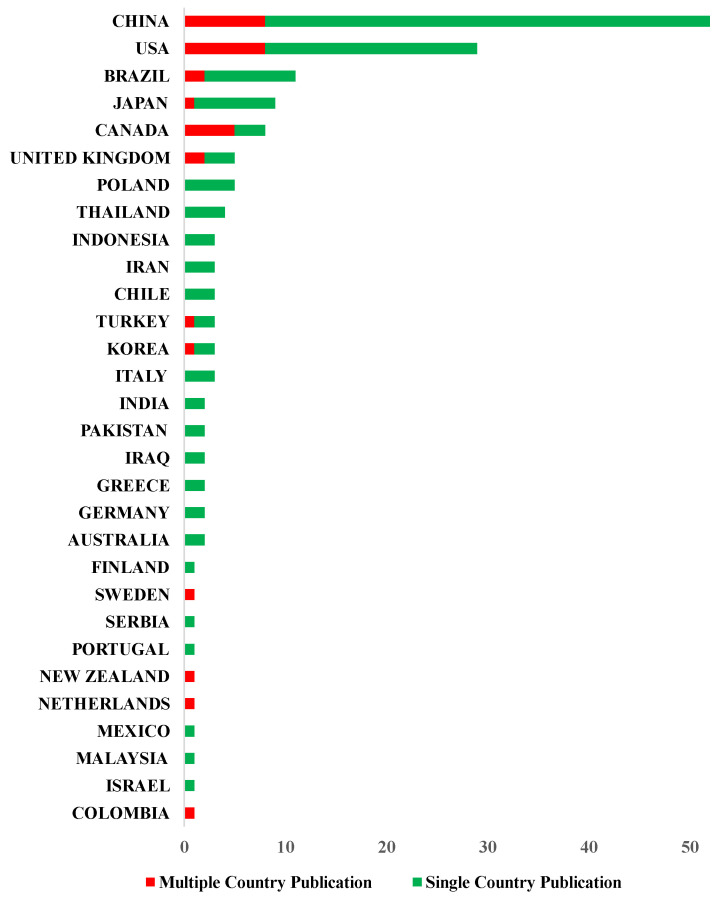
Number of publications on antimicrobial peptides for caries management according to country.

China (*n* = 52) and the US (*n* = 29) contributed almost half of the included publications (81/163, 49%). In addition, global collaborations for research on antimicrobial peptides for caries management were common in China (MCP/SCP = 8/44). Other counties such as the US (MCP/SCP = 8/29) and Canada (MCP/SCP = 8/29) were also involved in international collaborations (Figure 7).

The Sichuan University reported the greatest number of publications on antimicrobial peptides for caries management (*n* = 41), followed by the University of California, Los Angeles (*n* = 15); Sun Yat-sen University (*n* = 13); Federal University of Ceará (*n* = 12); Fourth Military Medical University (*n* = 11); and Seoul National University (*n* = 11).

## 4. Discussion

Antimicrobial peptides are a potential alternative to traditional antibiotics because of their selective toxicities, low chance of inducing resistance [20] and broad specificity [21,22]. In addition, antimicrobial peptides’ various and flexible structures allow the functional design and development of novel antimicrobial peptides [37]. An increasing number of researchers seek to be involved in antimicrobial peptides for caries management [8]. However, due to the enormous number of different antimicrobial peptides and the voluminous and fragmented body of research publications, it is not easy to understand and organise earlier findings in this field and begin new research [3,38]. Therefore, it is necessary to assess this field’s current research status. This study is the first bibliometric analysis of antimicrobial peptides for caries management. It provides an overview of the new progress of research on antimicrobial peptides for caries management and helps researchers identify the top scholars, institutions and journals on this topic.

Bibliometric analysis applies to all disciplines because it can help researchers understand previous findings and inspire new ideas [39]. However, bibliometric research is difficult because complex steps and numerous analyses and mapping software tools are required [40]. To address these challenges, Aria and Cuccurullo developed a comprehensive open-source tool, Bibliometrix, for performing bibliometric analysis. This flexible tool can be rapidly upgraded and integrated with other statistical R packages [23]. For this study, we selected three mainstream medical and dentistry databases (Web of Science, PubMed and Scopus) to identify publications for inclusion [41]. These databases provided complete citation information for the included publications. Based on these complete and reliable bibliometric data, a bibliometric analysis could be conducted using Bibliometrix.

This study included 163 publications for analysis. Most (*n* = 115) of the publications were laboratory studies. The citation counts of laboratory study publications were lower than were those of the review publications (no significant difference). However, the average citation density of reviews was significantly higher than that of laboratory studies and clinical trials. Citation density reflects the average number of citations each year [25]. A laboratory study on HBD-2 and HBD-3 against *S. mutans* and other oral pathogens obtained the highest number of citations [28]. This earlier finding demonstrated the antimicrobial properties against oral pathogens of antimicrobial peptides.

Among the other top five high-citation publications, three were laboratory studies and one was a review. Ouhara et al. investigated the antimicrobial properties against oral pathogens of another natural antimicrobial peptide, LL37 [29]. Wei et al. developed a series of antimicrobial peptides derived from mucins and tested their antimicrobial properties against oral pathogens [30]. Eckert et al. developed a novel specially targeted antimicrobial peptide, C16G2, against *S. mutans* [31]. This peptide was the only novel antimicrobial peptide processed for clinical trials [42]. In 2011, Batoni et al. reviewed antimicrobial peptides’ possible advantages and limits for their use against microbial biofilm-related infections [32].

A systematic review on the application of antimicrobial peptides for caries prevention and treatment obtained the highest average citation density, cited 21 times in one year [8]. This is the first systematic review on this topic. Qiu et al. and Mai et al. reviewed the use of antimicrobial peptides in dental caries and pulpal infections [3,33]. Xie et al. developed an antimicrobial peptide-polymer adhesive system against *S. mutans* [34].

Interestingly, different from four laboratory studies and a review among the top five high-citation counts publications, four reviews and one laboratory study constituted the top five high-citation density publications. Over time, older papers with earlier findings obtained more citations. In addition, a review with sufficient data/results and a persuasive language style could provide the readers substantial new innovative ideas [43]. These papers’ high citation records demonstrate that earlier findings and quality literature reviews are essential in a research field because they can help the researcher understand and become involved in a new research field [44].

Among the included publications were two main topics: novel antimicrobial peptides as anti-caries agents for caries management and natural antimicrobial peptides as biomarkers of caries risk. The laboratory studies mainly focused on novel antimicrobial peptides as anti-caries agents for caries management. All the publications used *S. mutans* as research subjects to investigate antimicrobial peptides’ antimicrobial properties. However, it should be noted that cariogenic species should not be limited to *S. mutans* [14]. Besides bacteria, fungi such as *Candida albicans* may contribute to the progressing of dental caries [8]. This is a major limitation of the studies on antimicrobial peptides for caries management. Some publications also investigated their novel antimicrobial peptides’ mineralising properties [45,46]. However, the major limitation of the studies is they use a chemical model which cannot reflect the role of bacteria in the demineralisation of tooth tissues. Some studies used single-specie biofilm [45,46]. To address these limitations, it is necessary to introduce multiple-species cariogenic biofilm [47] in future studies on antimicrobial peptides for caries management. In addition, only 8% of the studies deeply investigate the interaction mechanism of antimicrobial peptides with bacteria or tooth surfaces. We believe that the above-mentioned limitations reduced the studies’ quality and hindered them from processing into clinical trials. Among the laboratory studies, only one novel antimicrobial peptide, C16G2, was processed for clinical trial [42].

The other clinical trials mainly focused on natural antimicrobial peptides as biomarkers of caries risk. These studies observed some association between genetic polymorphisms of saliva antimicrobial peptides and risk of caries, but the definite relationship is still unclear [48,49]. In addition, the number of studies is limited. Researchers need to confirm whether antimicrobial peptides can serve as a biomarker of caries risk [49]. These research topics are consistent with the top keywords, such as ‘*Streptococcus mutans*’, ‘dental caries’, ‘antimicrobial peptides’, ‘biofilm’, ‘saliva’, and ‘mineralisation’, we detected from the bibliometric analysis.

According to Bradford’s law, ‘core’ journals are those that publish one-third of the publications in a particular field [26]. Interestingly, the list of ‘core’ journals differed slightly from the top h-index journals. There were 12 publications from the *Archives of Oral Biology*, but this journal’s h-index was 9. *Clinical Oral Investigations* is another journal in which the number of publications was higher than its h-index. The focus of Bradford’s law and that of the h-index is different. Bradford’s law focuses on the number of publications, whereas the h-index reflects the impact of the publications [26,27]. Thus, readers and future researchers interested in research on antimicrobial peptides for caries management should refer to both indexes.

The tools we used for this bibliometric analysis could determine both indexes simultaneously. Notably, from the list of journals determined as based on the indexes mentioned above, publications on antimicrobial peptides for caries management were published in dental journals and some molecular biology and microbiology journals. In addition, even in dental journals, most studies on antimicrobial peptides for caries management were only published in top middle journals such as *Archives of Oral Biology* and *Caries Research* [50]. This indicated that, although this topic has grown steadily hotter in the past two decades, the quality and impact of research on this topic still need to be increased.

This study also assessed the collaboration network of research on antimicrobial peptides in caries management worldwide. The scientific community encourages international co-authorship because publications resulting from international collaboration usually obtain higher citation counts and impacts [51,52]. However, China and the US contributed to almost half of the publications on antimicrobial peptides for caries management, and the MCP ratio was only 20%. A possible reason is that research in this field is still mainly in the early stages of laboratory study [8] and the emergence of drug-resistant bacteria necessitating the experimental research on novel antimicrobial peptides [53].

This study also had some limitations. We assessed the citation data of included publications. However, a high citation count does not suggest the publication’s high quality [54]. Citation data depend on the number of scholars in a particular field and can be manipulated [55]. In addition, we accepted self-citation in the present study.

Despite these limitations, we did a bibliometric analysis to offer a global overview of the extant trend of the research on antimicrobial peptides for caries management. The bibliometric analysis is necessary for readers and other researchers interested in this field. This bibliometric analysis indicated an increasing trend in global interest in antimicrobial peptides for caries management. Scholars majorly focus on the novel antimicrobial peptides as anti-caries agents for caries management and natural antimicrobial peptides as biomarkers of caries risk. The development of novel antimicrobial peptides is inspired by the ideal antimicrobial properties and flexible structures of natural antimicrobial peptides. Whereas the use of natural antimicrobial peptides as biomarkers of caries risk is inspired by the essential role of natural antimicrobial peptides in innate immunity. A series of laboratory, clinical, and literature reviews have been established. More and more new researchers have started to research this topic. However, the research on antimicrobial peptides for caries management still not does not break through the barriers from basic research to application in the actual world. That is why the review articles obtained more citations than that laboratory studies followed by clinical trials. In addition, the limited quality and quantity of studies limit the generation of high-quality evidence and clear conclusion. Overall, developing a standard research process for studying antimicrobial peptides for caries management is necessary. On the other hand, further pre-clinical and clinical trials should be conducted immediately after developing novel antimicrobial peptides. And multicentred studies with large sample sizes are also necessary for research on natural antimicrobial peptides as biomarkers of caries risk.

## 5. Conclusions

This bibliometric analysis identified an increasing trend in global interest in antimicrobial peptides for caries management since 2002. Development of novel antimicrobial peptides was the main research topic. Most publications were laboratory studies and they had high citation counts. The three publications with the highest citation counts were all laboratory studies. Reviews had high citation density among the publications.

## Figures and Tables

**Table 1:** Top five publications according to citation counts and citation density.

Authors, Year [Ref]	Study	Journal	Citation Counts	Citation Density
Top five publications with highest total citation counts				
Joly et al., 2004 [28]	Laboratory	*Journal of Clinical Microbiology*	205	11.39
Ouhara et al., 2005 [29]	Laboratory	*Journal of Antimicrobial Chemotherapy*	177	10.41
Wei et al., 2006 [30]	Laboratory	*Journal of Antimicrobial Chemotherapy*	163	10.19
Eckert et al., 2006 [31]	Laboratory	*Antimicrobial Agents and Chemotherapy*	158	9.88
Batoni et al., 2011 [32]	Review	*Current Medicinal Chemistry*	137	12.45
Top five publications with highest citation density				
Niu et al., 2021 [8]	Review	*Archives of Oral Biology*	21	21.00
Qiu et al., 2020 [33]	Review	*Biomed Research International*	27	13.50
Mai et al., 2017 [3]	Review	*Acta Biomaterialia*	67	13.40
Batoni et al., 2011 [32]	Review	*Current Medicinal Chemistry*	137	12.45
Xie er al., 2020 [34]	Laboratory	*Acs Applied Polymer Materials*	24	12.00
*Citation density* = *Citation count*/(2022 − *published year*)				

**Table 2:** Number of publications, citation count and citation density according to type of study.

Study	Number (%)	Mean Citation Count (SD)	Mean Citation Density (SD)
Laboratory	115 (71%)	29 (36)	3.67 (2.73)
Clinical	29 (18%)	22 (27)	2.63 (1.85)
Review	19 (11%)	31 (34)	5.79 (1.27)
*p* value		0.560	0.002

## Data Availability

Not applicable.

## Supplementary Materials

The following supporting information can be downloaded at: https://www.mdpi.com/article/10.3390/jfb13040210/s1, Supplementary Table S1. Information of the included publications.
