# Supplementary material for: Growing Global Research Interest in Antimicrobial Peptides for Caries Management: A Bibliometric Analysis
Source: J Funct Biomater. 2022 Oct 29;13(4):210. doi: 10.3390/jfb13040210 (PMC9680375; doi:10.3390/jfb13040210)
Supplement: Supplementary file 1 [file jfb-13-00210-s001.zip › jfb-1998142-supplementary.pdf]

Supplementary Materials

Growing Global Research Interest in Antimicrobial Peptides for Caries Management: A Bibliometric Analysis

Olivia Lili Zhang [1], John Yun Niu [1], Iris Xiaoxue Yin [1], Ollie Yiru Yu [1], May Lei Mei [2] and Chun Hung Chu [1,*]

[1] Faculty of Dentistry, The University of Hong Kong, Hong Kong, China
[2] Faculty of Dentistry, The University of Otago, Dunedin 9054, New Zealand
* Correspondence: chchu@hku.hk

Table S1. Information of the included publications

Title	Source	Publication Year	Total Citation	Citation Density
Antimicrobial peptides for the prevention and treatment of dental caries: A concise review	Archives of Oral Biology	2021	21	21.00
Application of Antibiotics/Antimicrobial Agents on Dental Caries	Biomed Research International	2020	27	13.50
Potential applications of antimicrobial peptides and their mimics in combating caries and pulpal infections	Acta Biomaterialia	2017	67	13.40
Use of Antimicrobial Peptides Against Microbial Biofilms: Advantages and Limits	Current Medicinal Chemistry	2011	137	12.45
Antimicrobial Peptide-Polymer Conjugates for Dentistry	Acs Applied Polymer Materials	2020	24	12.00
Precision-guided antimicrobial peptide as a targeted modulator of human microbial ecology	Proceedings of the National Academy of Sciences of the United States of America	2015	81	11.57
Human beta-defensins 2 and 3 demonstrate strain-selective activity against oral microorganisms	Journal of Clinical Microbiology	2004	205	11.39
Susceptibilities of periodontopathogenic and cariogenic bacteria to antibacterial peptides, β-defensins and LL37, produced by human epithelial cells	Journal of Antimicrobial Chemotherapy	2005	177	10.41
Effect of MUC7 peptides on the growth of bacteria and on Streptococcus mutans biofilm	Journal of Antimicrobial Chemotherapy	2006	163	10.19

Inhibitory effect of LL-37 and human lactoferricin on growth and biofilm formation of anaerobes associated with oral diseases	Anaerobe	2021	10	10.00
Targeted killing of Streptococcus mutans by a pheromone-guided "smart" antimicrobial peptide	Antimicrobial Agents and Chemotherapy	2006	158	9.88
Antimicrobial peptide GH12 suppresses cariogenic virulence factors of Streptococcus mutans	Journal of Oral Microbiology	2018	34	8.50
Antimicrobial peptide-loaded liquid crystalline precursor bioadhesive system for the prevention of dental caries	International Journal of Nanomedicine	2018	33	8.25
Treatment of Oral Multispecies Biofilms by an Anti-Biofilm Peptide	Plos One	2015	57	8.14
Activity of an antimicrobial peptide mimetic against planktonic and biofilm cultures of oral pathogens	Antimicrobial Agents and Chemotherapy	2007	113	7.53
Salivary antimicrobial peptide expression and dental caries experience in children	Antimicrobial Agents and Chemotherapy	2005	128	7.53
Constructing an Antibiofouling and Mineralizing Bioactive Tooth Surface to Protect against Decay and Promote Self-Healing	Acs Applied Materials & Interfaces	2020	15	7.50
De novo synthetic short antimicrobial peptides against cariogenic bacteria	Archives of Oral Biology	2017	37	7.40
Gene-environment Interactions in the Etiology of Dental Caries	Journal of Dental Research	2016	44	7.33
Antibacterial peptide nisin: A potential role in the inhibition of oral pathogenic bacteria	Peptides	2014	57	7.13
Antimicrobial and anti-biofilm effect of Bac8c on major bacteria associated with dental caries and Streptococcus mutans biofilms	Peptides	2014	55	6.88
Antimicrobial peptide control of pathogenic microorganisms of the oral cavity: A review of the literature	Peptides	2012	68	6.80

Antibiofilm peptides against biofilms on titanium and hydroxyapatite surfaces	Bioactive Materials	2018	26	6.50
Effect of a novel antimicrobial peptide chrysophsin-1 on oral pathogens and Streptococcus mutans biofilms	Peptides	2012	62	6.20
Effects of antimicrobial peptide L-K6, a temporin-1CEb analog on oral pathogen growth, Streptococcus mutans biofilm formation, and anti-inflammatory activity	Applied Microbiology and Biotechnology	2014	49	6.13
A tooth-binding antimicrobial peptide to prevent the formation of dental biofilm	Journal of Materials Science-Materials in Medicine	2019	18	6.00
Cationic Antimicrobial Peptides Are Leading the Way to Combat Oropathogenic Infections	Acs Infectious Diseases	2021	6	6.00
A Novel Strategy for Caries Management: Constructing an Antibiofouling and Mineralizing Dual-Bioactive Tooth Surface	Acs Applied Materials & Interfaces	2021	6	6.00
Antibacterial effect and bond strength of a modified dental adhesive containing the peptide nisin	Peptides	2018	22	5.50
The Antimicrobial Peptide DEFB1 Is Associated with Caries	Journal of Dental Research	2010	65	5.42
Nisin inhibits dental caries-associated microorganism in vitro	Peptides	2010	65	5.42
Design and antimicrobial activities of LL-37 derivatives inhibiting the formation of Streptococcus mutans biofilm	Chemical Biology & Drug Design	2019	16	5.33
In vitro assessment of antimicrobial peptides as potential agents against several oral bacteria	Journal of Antimicrobial Chemotherapy	2006	85	5.31
Selective Membrane Disruption: Mode of Action of C16G2, a Specifically Targeted Antimicrobial Peptide	Antimicrobial Agents and Chemotherapy	2011	56	5.09
Promotion of enamel caries remineralization by an amelogenin-derived peptide in a rat model	Archives of Oral Biology	2017	25	5.00
Antimicrobial peptide GH12 targets Streptococcus mutans to arrest caries development in rats	Journal of Oral Microbiology	2019	15	5.00

Bifunctional anticaries peptides with antibacterial and remineralizing effects	Oral Diseases	2019	15	5.00
Chitosan hydrogel containing amelogenin-derived peptide: Inhibition of cariogenic bacteria and promotion of remineralization of initial caries lesions	Archives of Oral Biology	2019	15	5.00
A Novel Target-Specific, Salt-Resistant Antimicrobial Peptide against the Cariogenic Pathogen Streptococcus mutans	Antimicrobial Agents and Chemotherapy	2011	54	4.91
Potential of an amelogenin based peptide in promoting reminerlization of initial enamel caries	Archives of Oral Biology	2015	34	4.86
Activity of Synthetic Antimicrobial Peptide GH12 against Oral Streptococci	Caries Research	2016	29	4.83
Treatment of Oral Biofilms by a D-Enantiomeric Peptide	Plos One	2016	29	4.83
Antimicrobial and DNA-binding activities of the peptide fragments of human lactoferrin and histatin 5 against Streptococcus mutans	Archives of Oral Biology	2011	52	4.73
Antibacterial and Antibiofilm Activities of a Novel Synthetic Cyclic Lipopeptide against Cariogenic Streptococcus mutans UA159	Antimicrobial Agents and Chemotherapy	2017	23	4.60
A universal adhesive incorporating antimicrobial peptide nisin: effects on Streptococcus mutans and saliva-derived multispecies biofilms	Odontology	2020	9	4.50
Oral antimicrobial peptides and biological control of caries	BMC Oral Health	2006	71	4.44
Antibiofilm peptides against oral biofilms	Journal of Oral Microbiology	2017	22	4.40
Salivary concentration of the antimicrobial peptide LL-37 in children	Archives of Oral Biology	2012	43	4.30
Design and Characterization of an Acid-Activated Antimicrobial Peptide	Chemical Biology & Drug Design	2010	51	4.25

Effects of Antimicrobial Peptide GH12 on the Cariogenic Properties and Composition of a Cariogenic Multispecies Biofilm	Applied and Environmental Microbiology	2018	17	4.25
A synthetic peptide adhesion epitope as a novel antimicrobial agent	Current Pharmaceutical Design	1999	96	4.17
Antimicrobial peptides in saliva of children with severe early childhood caries	Archives of Oral Biology	2016	25	4.17
Activity of human beta-defensin 3 alone or combined with other antimicrobial agents against oral bacteria	Antimicrobial Agents and Chemotherapy	2003	78	4.11
Clinical Efficacy of a Specifically Targeted Antimicrobial Peptide Mouth Rinse: Targeted Elimination of Streptococcus mutans and Prevention of Demineralization	Caries Research	2011	44	4.00
A novel hydroxyapatite-binding antimicrobial peptide against oral biofilms	Clinical Oral Investigations	2019	12	4.00
Targeting the oral plaque microbiome with immobilized anti-biofilm peptides at tooth-restoration interfaces	Plos One	2020	8	4.00
Beta-defensin 1 gene polymorphisms in the pathologies of the oral cavity-Data from meta-analysis: Association only with rs1047031 not with rs1800972, rs1799946, and rs11362	Journal of Oral Pathology & Medicine	2021	4	4.00
The multifaceted roles of antimicrobial peptides in oral diseases	Molecular Oral Microbiology	2021	4	4.00
TVH-19, a synthetic peptide, induces mineralization of dental pulp cells in vitro and formation of tertiary dentin in vivo	Biochemical and Biophysical Research Communications	2021	4	4.00
Effects of a derivative of reutericin 6 and gassericin A on the biofilm ofStreptococcus mutansin vitro and caries prevention in vivo	Odontology	2021	4	4.00

The pH-Responsive Property of Antimicrobial Peptide GH12 Enhances Its Anticaries Effects at Acidic pH	Caries Research	2021	4	4.00
Antimicrobial and antibiofilm activity of pleurocidin against cariogenic microorganisms	Peptides	2011	43	3.91
Divalent metal cations increase the activity of the antimicrobial peptide kappacin	Antimicrobial Agents and Chemotherapy	2005	66	3.88
Systematic Approach to Optimizing Specifically Targeted Antimicrobial Peptides against Streptococcus mutans	Antimicrobial Agents and Chemotherapy	2010	46	3.83
Effect of the antimicrobial decapeptide KSL on the growth of oral pathogens and Streptococcus mutans biofilm	International Journal of Antimicrobial Agents	2011	42	3.82
Targeted antimicrobial treatment to re-establish a healthy microbial flora for long-term protection	Advances in dental research	2012	38	3.80
A novel antimicrobial peptide against dental-caries-associated bacteria	Anaerobe	2017	19	3.80
Enhancement of salivary human neutrophil peptide 1-3 levels by probiotic supplementation	Bmc Oral Health	2015	26	3.71
The role of salivary peptides in dental caries	Biomedical Chromatography	2005	63	3.71
Design of a hydroxyapatite-binding antimicrobial peptide with improved retention and antibacterial efficacy for oral pathogen control	Scientific Reports	2016	22	3.67
Anti-biofilm and anti-adherence properties of novel cyclic dipeptides against oral pathogens	Bioorganic and Medicinal Chemistry	2019	11	3.67
Antimicrobial activity of the synthetic peptide Lys-a1 against oral streptococci	Peptides	2013	32	3.56
Design and activity of a 'dual-targeted' antimicrobial peptide	International Journal of Antimicrobial Agents	2009	46	3.54
Cytotoxicity and the effect of cationic peptide fragments against cariogenic bacteria under planktonic and biofilm conditions	Biofouling	2016	21	3.50

Temporin-Like Peptides Show Antimicrobial and Anti-Biofilm Activities against Streptococcus mutans with Reduced Hemolysis	Molecules	2020	7	3.50
Antimicrobial Peptide GH12 Prevents Dental Caries by Regulating Dental Plaque Microbiota	Applied and Environmental Microbiology	2020	7	3.50
Antibacterial Efficacy of a Human beta-Defensin-3 Peptide on Multispecies Biofilms	Journal of Endodontics	2013	31	3.44
A study on beta-defensin-2 and histatin-5 as a diagnostic marker of early childhood caries progression	Biological Research	2015	24	3.43
The synthetic human beta-defensin-3 C15 peptide exhibits antimicrobial activity against Streptococcus mutans, both alone and in combination with dental disinfectants	Journal of Microbiology	2017	17	3.40
Novel synthetic antimicrobial peptides against Streptococcus mutans	Antimicrobial Agents and Chemotherapy	2007	49	3.27
A statherin-derived peptide promotes hydroxyapatite crystallization and in situ remineralization of artificial enamel caries	Rsc Advances	2018	13	3.25
Salivary protein polymorphisms and risk of dental caries: a systematic review	Brazilian Oral Research	2017	16	3.20
Antimicrobial Activity and Mechanism of Action of a Novel Cationic alpha-Helical Octadecapeptide Derived From alpha-Amylase of Rice	Biopolymers	2015	22	3.14
Streptococcus mutans strains recovered from caries-active or caries-free individuals differ in sensitivity to host antimicrobial peptides	Molecular Oral Microbiology	2011	34	3.09
Targeted Antimicrobial Therapy Against Streptococcus mutans Establishes Protective Non-cariogenic Oral Biofilms and Reduces Subsequent Infection	International Journal of Oral Science	2010	37	3.08

Bioinspired Peptide-Decorated Tannic Acid for in Situ Remineralization of Tooth Enamel: In Vitro and in Vivo Evaluation	Acs Biomaterials Science & Engineering	2017	15	3.00
Combined analysis of the salivary microbiome and host defence peptides predicts dental disease	Scientific Reports	2018	12	3.00
Antimicrobial Effect of a Peptide Containing Novel Oral Spray on Streptococcus mutans	Biomed Research International	2020	6	3.00
A single system detects and protects the beneficial oral bacterium Streptococcus sp. A12 from a spectrum of antimicrobial peptides	Molecular Microbiology	2021	3	3.00
Novel lactotransferrin-derived synthetic peptides suppress cariogenic bacteria in vitro and arrest dental caries in vivo	Journal of Oral Microbiology	2021	3	3.00
The consumption of milk supplemented with probiotics decreases the occurrence of caries and the salivary concentration of h beta D-3 in children	Clinical Oral Investigations	2021	3	3.00
Significant elevation of salivary human neutrophil peptides 1-3 levels by probiotic milk in preschool children with severe early childhood caries: a randomized controlled trial	Clinical Oral Investigations	2021	3	3.00
Antibacterial Peptides: Opportunities for the Prevention and Treatment of Dental Caries	Probiotics and Antimicrobial Proteins	2011	32	2.91
Evaluation of antibacterial, antibofilm and antioxidant activities of synthesized silver nanoparticles (AgNPs) and casein peptide fragments against Streptococcus mutans	European Journal of Integrative Medicine	2017	14	2.80
Peptide pheromone induced cell death of Streptococcus mutans	Fems Microbiology Letters	2005	47	2.76
The assessment of sIgA, histatin-5, and lactoperoxidase levels in saliva of adolescents with dental caries	Medical Science Monitor	2014	22	2.75

Peptide and non-peptide mimetics as potential therapeutics targeting oral bacteria and oral biofilms	Molecular Oral Microbiology	2019	8	2.67
Correlation between human neutrophil peptide 1-3 secretion and azurophilic granule (CD63) expression in early childhood caries	Dental Research Journal	2019	8	2.67
Development of a peptide-containing chewing gum as a sustained release antiplaque antimicrobial delivery system	Aaps Pharmscitech	2007	39	2.60
An in vitro synergetic evaluation of the use of nisin and sodium fluoride or chlorhexidine against Streptococcus mutans	Peptides	2011	28	2.55
Statherin and histatin 1 reduce parotid saliva-promoted Streptococcus mutans strain MT8148 adhesion to hydroxyapatite surfaces	Caries Research	2006	40	2.50
Salivary antimicrobial proteins associate with age-related changes in streptococcal composition in dental plaque	Molecular Oral Microbiology	2014	20	2.50
The investigation of synergistic activity of protamine with conventional antimicrobial agents against oral bacteria	Biochemical and Biophysical Research Communications	2020	5	2.50
Salivary peptide profile and its association with early childhood caries	International Journal of Paediatric Dentistry	2013	21	2.33
Effect of histatin-5 and lysozyme on the ability of Streptococcus mutans to form biofilms in vitro conditions	Postepy Higieny I Medycyny Doswiadczalnej	2015	16	2.29
Selective activities of STAMPs against Streptococcus mutans	Experimental and Therapeutic Medicine	2018	9	2.25
Sensitivity of caries pathogens to antimicrobial peptides related to caries risk	Clinical Oral Investigations	2018	9	2.25
Antibacterial activity of a novel antimicrobial peptide [W7]KR12-KAEK derived from KR-12 against Streptococcus mutans planktonic cells and biofilms	Biofouling	2017	11	2.20

Genetic Polymorphisms in DEFB1 and miRNA202 Are Involved in Salivary Human beta-Defensin 1 Levels and Caries Experience in Children	Caries Research	2017	11	2.20
Genetic variation in the promoter region of beta-defensin 1 (DEFB 1) is associated with high caries experience in children born with cleft lip and palate	Acta Odontologica Scandinavica	2014	17	2.13
Oral streptococci exhibit diverse susceptibility to human beta-defensin 2: Antimicrobial effects of hBD-2 on oral streptococci	Current Microbiology	2004	38	2.11
Endotoxin-neutralizing activity and mechanism of action of a cationic alpha-helical antimicrobial octadecapeptide derived from alpha-amylase of rice	Peptides	2016	12	2.00
Caries and Innate Immunity: DEFB1 Gene Polymorphisms and Caries Susceptibility in Genetic Isolates from North-Eastern Italy	Caries Research	2016	12	2.00
Duplicated or Hybridized Peptide Functional Domains Promote Oral Homeostasis	Journal of Dental Research	2017	10	2.00
A novel non-cytotoxic synthetic peptide, Pug-1, exhibited an antibiofilm effect on Streptococcus mutans adhesion	Letters in Applied Microbiology	2020	4	2.00
A novel dual-action antimicrobial peptide for caries management	Journal of Dentistry	2021	2	2.00
Suppressive effects of gecko cathelicidin on biofilm formation and cariogenic virulence factors of Streptococcus mutans	Archives of Oral Biology	2021	2	2.00
Downregulation of Salivary Proteins, Protective against Dental Caries, in Type 1 Diabetes	Proteomes	2021	2	2.00
Susceptibility of periodontopathogenic and cariogenic bacteria to Defensins and potential therapeutic use of Defensins in oral diseases	Current Pharmaceutical Design	2007	29	1.93
In vitro antimicrobial effect of bacteriocin PsVP-10 in combination with chlorhexidine and triclosan against	Archives of Oral Biology	2009	23	1.77

Streptococcus mutans and Streptococcus sobrinus strains				
A synthetic peptide based on a natural salivary protein reduces demineralisation in model systems for dental caries and erosion	International Journal of Peptide Research and Therapeutics	2007	25	1.67
Rational design of peptides with enhanced antimicrobial and anti-biofilm activities against cariogenic bacterium Streptococcus mutans	Chemical Biology & Drug Design	2019	5	1.67
Streptococcal adhesin SspA/B analogue peptide inhibits adherence and impacts biofilm formation of Streptococcus mutans	Plos One	2017	8	1.60
Effect of the Antimicrobial Peptide D-Nal-Pac-525 on the Growth of Streptococcus mutans and Its Biofilm Formation	Journal of Microbiology and Biotechnology	2013	14	1.56
Association between genetic polymorphisms in DEFB1 and microRNA202 with caries in two groups of Brazilian children	Archives of Oral Biology	2018	6	1.50
Salivary proteins and peptides in the aetiology of caries in children: Systematic literature review	Oral Diseases	2019	4	1.33
A Membrane-Targeted Peptide Inhibiting PtxA of Phosphotransferase System Blocks Streptococcus mutans	Caries Research	2019	4	1.33
Inhibition of Streptococcus mutans adherence and biofilm formation using analogues of the SspB peptide	Archives of Oral Biology	2010	15	1.25
Host-derived pentapeptide affecting adhesion, proliferation, and local pH in biofilm communities composed of Streptococcus and Actinomyces species	Infection and Immunity	2006	19	1.19
Remineralization of initial enamel caries in vitro using a novel peptide based on amelogenin	Frontiers of Materials Science	2015	8	1.14
Salivary mucin as related to oral Streptococcus mutans in elderly people	Oral Microbiol Immunol	2000	24	1.09

Design of a peptibody consisting of the antimicrobial peptide dhvar5 and a llama variable heavy-chain antibody fragment	Chemical Biology & Drug Design	2006	17	1.06
The impact of salivary lactoperoxidase and histatin-5 on early childhood caries severity in relation to nutritional status	Saudi Dental Journal	2020	2	1.00
Dual-sensitive antibacterial peptide nanoparticles prevent dental caries	Theranostics	2022	1	1.00
Remineralising dentine caries using an artificial antimicrobial peptide: An in vitro study	Journal of Dentistry	2021	1	1.00
Bioinspired caries preventive strategy via customizable pellicles of saliva-derived protein/peptide constructs	Scientific Reports	2021	1	1.00
Influence of Gallic Acid and Thai Culinary Essential Oils on Antibacterial Activity of Nisin against Streptococcus mutans	Advances in Pharmacological and Pharmaceutical Sciences	2021	1	1.00
Study of the ultrastructure of Enterococcus faecalis and Streptococcus mutans incubated with salivary antimicrobial peptides	Clinical and Experimental Dental Research	2021	1	1.00
The Association of Early Childhood Caries with Salivary Antimicrobial Peptide LL37 and Mutans Streptococci	Journal of Clinical Pediatric Dentistry	2021	1	1.00
The Relation of Salivary Cathelicidin and Beta-Defensin with Dental Caries of Schoolchildren	Journal of Research in Medical and Dental Science	2021	1	1.00
Factors affecting antimicrobial activity of MUC7 12-mer, a human salivary mucin-derived peptide	Annals of Clinical Microbiology and Antimicrobials	2007	14	0.93
Synergistic inhibitory effect of cationic peptides and antimicrobial agents on the growth of oral streptococci	Caries Research	2003	17	0.89
The level of beta defensin-2 in saliva and its expression in parotid gland epithelial cells after	European Journal of Dentistry	2016	5	0.83

probiotic (Lactobacillus reuteri) induction to inhibit Streptococcus mutans in caries				
New Preventive Approaches Part I: Functional Peptides and Other Therapies to Prevent Tooth Demineralization	Monographs in Oral Science	2017	3	0.60
In vitro antibacterial activity of the peptide PsVP-10 against Streptococcus mutans and Streptococcus sobrinus with and without glycocalyx	International Journal of Antimicrobial Agents	2006	9	0.56
Association between rs11362 polymorphism in the beta-defensin 1 (DEFB1) gene and dental caries: A meta-analysis	Journal of Oral Biosciences	2020	1	0.50
Can Salivary Biomarkers Be Used as Predictors of Dental Caries in Young Adolescents?	Medical Science Monitor	2020	1	0.50
Salivary lysozyme, lactoferrin and peroxidases: antibacterial effects on cariogenic bacteria and clinical applications in preventive dentistry	Proceedings of the Finnish Dental Society. Suomen Hammaslaakariseuran toimituksia	1991	14	0.45
Peptide inhibitors of Streptococcus mutans in the control of dental caries	International Journal of Peptide Research and Therapeutics	2007	4	0.27
Effects of cecropin-XJ on growth and adherence of oral cariogenic bacteria in vitro	Chinese Medical Journal	2005	3	0.18
Peptide-Enabled Nanocomposites Offer Biomimetic Reconstruction of Silver Diamine Fluoride-Treated Dental Tissues	Polymers (Basel)	2022	0	0.00
Efficacy of the dual-action GA-KR12 peptide for remineralising initial enamel caries: an in vitro study	Clinical Oral Investigations	2022	0	0.00
Engineered Salivary Peptides Reduce Enamel Demineralization Provoked by Cariogenic S. mutans Biofilm	Microorganisms	2022	0	0.00
Constructing an anti-S. mutans and mineralizing membrane by combination self-assembled lysozyme with antimicrobial peptide	Materials and Design	2022	0	0.00

The Application of a Recombinant Antimicrobial Peptide Thrombocidin-1 Expressed in Pichia pastoris as a Novel Approach Against Some Oral Pathogenic Bacteria: An In Vitro Study	Protein and Peptide Letters	2022	0	0.00
Selective antibacterial activity of a novel lactotransferrin-derived antimicrobial peptide LF-1 against Streptococcus mutans	Archives of Oral Biology	2022	0	0.00
Quantitative analysis of Protective Role of salivary protein on demineralization of enamel	Pakistan Journal of Medical & Health Sciences	2021	0	0.00
Evaluation of possible biomarkers for caries risk in children 6 to 12 years of age	Journal of Oral Microbiology	2021	0	0.00
Spectrophotometric Analysis of Streptococcus mutans Growth and Biofilm Formation in Saliva and Histatin-5 Relate to pH and Viscosity	Pesquisa Brasileira Em Odontopediatria E Clinica Integrada	2021	0	0.00
A New Approach Against Some Oral Pathogenic Bacteria Using a Chimeric Antimicrobial Peptide Derived from the Camel Milk; Lactoferrampin Lactoferricin Chimer	Current Drug Discovery Technologies	2021	0	0.00
Associations of early childhood caries with salivary beta defensin-3 and childhood anemia: a case-control study	Bmc Oral Health	2021	0	0.00
Association of β-defensin 1 gene polymorphism and dental caries susceptibility in tamil ethnicity	Research Journal of Pharmacy and Technology	2021	0	0.00
Salivary peptide human neutrophil defensin1-3 and its relationship with early childhood caries	Dental Research Journal	2020	0	0.00
